# Phytochemical characterization and antifungal activity of medicinal plant extracts against *Colletotrichum* species associated with anthracnose in Thailand

**DOI:** 10.1371/journal.pone.0339399

**Published:** 2025-12-30

**Authors:** Nittaya Pitiwittayakul, Nanthavut Niyomvong, Duanpen Wongsorn, Jaturong Kumla, Nakarin Suwannarach

**Affiliations:** 1 Department of Plant Science, Faculty of Agricultural Innovation and Technology, Rajamangala University of Technology Isan, Nakhon Ratchasima campus, Nakhon Ratchasima, Thailand; 2 Science Center, Nakhon Sawan Rajabhat University, Nakhon Sawan, Thailand; 3 Department of Biology and Biotechnology, Faculty of Science and Technology, Nakhon Sawan Rajabhat University, Nakhon Sawan, Thailand; 4 Office of Research Administration, Chiang Mai University, Chiang Mai, Thailand; 5 Center of Excellence in Microbial Diversity and Sustainable Utilization, Chiang Mai University, Chiang Mai, Thailand; 6 Department of Biology, Faculty of Science, Chiang Mai University, Chiang Mai, Thailand; ICAR National Bureau of Agriculturally Important Microorganism, INDIA

## Abstract

*Colletotrichum* is a major plant-pathogenic fungus responsible for anthracnose, a disease that significantly reduces crop yield and quality, thereby hindering commercial production. This study aimed to isolate, identify, and characterize *Colletotrichum* species associated with anthracnose in various fruits and ornamental plants in Thailand, using both morphological and molecular approaches. Subsequently, the medicinal plant extracts were evaluated for their antifungal activity against the isolated *Colletotrichum* species, and their bioactive compounds were profiled using GC-MS and LC-MS analyses. Eleven *Colletotrichum* isolates were obtained and identified as nine distinct species, namely *C. asianum*, *C. brasiliense*, *C. fructicola*, *C. musae*, *C. nymphaeae*, *C. okinawense*, *C. orchidearum*, *C. pandanicola*, and *C. truncatum*. Among the ten medicinal plants tested, only the ethanolic extract of clove (*Syzygium aromaticum*) exhibited strong antifungal activity against all fungal isolates. The extract exhibited minimum inhibitory concentrations as low as 12.5 mg/mL against *C. okinawense* and *C. orchidearum*, and 25 mg/mL against the remaining species. GC-MS profiling of the ethanolic clove extract revealed that 2-methoxy-4-prop-2-enylphenol (eugenol, 72.417%) was the predominant compound, followed by (1R,4E,9S)-4,11,11-trimethyl-8-methylenebicyclo [7.2.0] undec-4-ene (β-caryophyllene, 12.125%), and (2-methoxy-4-prop-2-enylphenyl) acetate (eugenyl acetate, 8.121%). Additionally, LC-MS profiling indicated that the extract contained several antifungal constituents, including quercetin, kaempferol, isorhamnetin, myricetin, gallic acid, ellagic acid, catechol, caffeic acid, *p*-coumaric acid, methyl trans-cinnamic acid, and resveratrol. These findings highlight the diversity among pathogenic *Colletotrichum* species in Thailand and establish that clove extract, which contains flavonoids, phenolics, terpenoids, and alkaloids, holds potential as an eco-friendly alternative for agricultural disease management.

## Introduction

*Colletotrichum* is a genus of economically significant ascomycetous fungi that collectively cause anthracnose and leaf blight diseases in major agricultural crops and ornamental plants worldwide [1–4]. Infections primarily occur in tropical and subtropical regions, while they are less common in temperate areas due to their dependence on warm temperatures and high relative humidity [5]. Anthracnose is a severe disease affecting climacteric fruits such as avocados, bananas, guavas, mangoes, papayas, and pears, largely due to the physiological and biochemical changes that occur during ripening. These changes including cell wall remodeling and degradation create favorable conditions for fungal growth and pose a significant threat to non-climacteric fruits such as dragon fruit, oranges, and strawberries [6]. In fruit crops, this disease can lead to significant reductions in yield and quality, ultimately causing customer dissatisfaction and economic losses.

Traditional methods of controlling *Colletotrichum*, the causal agent of anthracnose in several fruits, have involved the use of various synthetic fungicides, such as mancozeb, carbendazim, thiram, ziram, captan, prochloraz, and tiabendazole [7]. However, the extensive use of chemical fungicides has contributed to the emergence of fungicide-resistant strains in *Colletotrichum* species, reducing treatment efficacy and complicating disease management [8,9]. In addition, prolonged fungicide application poses risks to food safety, the environment, and human health. In response to these concerns, green agricultural technologies for plant pathogen control are being increasingly promoted due to their ecological, safety, and societal benefits. Plants serve as rich sources of bioactive compounds, including terpenes, phenolics, essential oils, and alkaloids [10,11]. Thailand, in particular, possesses a high diversity of medicinal plants, many of which have been reported for their therapeutic properties [12]. Several studies have demonstrated that plant extracts contain a wide array of bioactive compounds with antifungal properties, making them promising alternatives to synthetic fungicides [13–16]. Numerous essential oils, alkane hydrocarbons, and fatty acids from various plant extracts have been identified using gas chromatography-mass spectrometry (GC-MS), while polyphenols and triterpenoids have been identified through liquid chromatography-mass spectrometry (LC-MS) analyses [17–19]. For a comprehensive phytochemical characterization, GC-MS and LC-MS are frequently employed in tandem, as their complementary analytical capabilities enable the detection of a broad spectrum of compounds in plant extracts. Therefore, this study aims to isolate *Colletotrichum* from tropical fruits and ornamental plants exhibiting anthracnose symptoms in Thailand, and to identify the isolates using both morphological characteristics and multi-gene molecular techniques. Furthermore, the antifungal activity of selected medicinal plant extracts was evaluated, and the most effective extract was characterized for its chemical composition using GC-MS and LC-MS analyses.

## Materials and methods

### Anthracnose sample collection

A total of 11 diseased plant samples showing anthracnose symptoms, representing nine plant species, were surveyed and collected in Thailand between 2023–2024. These included two samples from mango fruits (*Mangifera indica* L. cv. ‘Nam Dok Mai’ and *M. indica* L. cv. ‘Kaew Kamin’), one sample from a mango leaf, two samples from avocados (*Persea americana* Mill), and one sample each from chili [*Capsicum annuum* L. (Syn. *Capsicum frutescens* L. var. *frutescens*)], strawberry (*Fragaria* × *ananassa* Duchesne), passion fruit (*Passiflora edulis* Sims), Holland papaya (*Carica papaya* L.), banana (*Musa* ×* sapientum* L. cv. ‘Nam Wa’), and a Strawberry Shake philodendron leaf (*Philodendron erubescens* cv. ‘Strawberry Shake’). The samples were collected from local markets in Chiang Mai Province, Thailand, except for the ornamental plant Strawberry Shake philodendron, which was collected in Nakhon Ratchasima Province.

### Fungal isolation and morphological study

Naturally infected plant parts and fruits showing typical anthracnose lesions were incubated at 25°C for 1–2 days in a plastic container lined with moist filter paper to encourage sporulation. When spore masses or conidiomata appeared, single-spore isolation was performed according to Choi et al. [20]. Using a sterile Pasteur pipette, a drop of the conidial suspension in sterile 0.85% NaCl solution was dragged across the surface of 2% water agar to separate individual conidia. After 24–48 h of incubation at 25°C, germinating spores were observed under a microscope, and single germinated spores were transferred to potato dextrose agar (PDA; Corda, Madrid, Spain) supplemented with 0.5 mg/L streptomycin. Pure cultures of each fungal isolate were obtained through successive subculturing on PDA. Pure fungal isolates were maintained short-term on PDA slants and preserved long-term in 20% glycerol at −80°C. Colony morphology including colony shape, color, and pigmentation was determined. The following micromorphological characteristics were examined: conidiomata and conidiophores. The mean lengths and width of 50 randomly selected conidia from each isolate were measured using 100X magnification in a microscope (Nikon Ti-S inverted microscope, Japan). Each pure fungal culture was deposited in the Culture Collection of the Sustainable Development of Biological Resources (SDBR) Laboratory, Faculty of Science, Chiang Mai University (CMU), Thailand.

### DNA extraction and PCR amplification

DNA was extracted from a one-week-old pure culture grown on PDA. Genomic DNA was obtained using the DNA Extraction Mini Kit (FAVORGEN, Taiwan), following the manufacturer’s instructions. The targeted gene regions, primers, and PCR thermal cycle programs used for amplification are detailed in Table 1. Each PCR amplification reaction had a total volume is 20 μL, consisting of 6 μL ddH_2_O, 10 μL 2x Quick TaqTM HS DyeMix (TOYOBO, Japan), 2 μL DNA template, and 1 μL each of forward and reverse primers [21]. PCR products were purified using the PrimeWay Gel Extraction/ PCR Extraction Kit (1^st^Base, Malaysia) and directly sequenced via Sanger sequencing by 1^st^ Base Company (Kembangan, Malaysia) using the same primers mentioned above.

**Table 1:** Primer used in this study.

Gene	Primers	PCR conditions	References
ITS	ITS4/ITS5	95 °C: 2 mins, (95 °C: 30 s, 52 °C: 30 s, 72 °C: 60 s) × 35 cycles, 72 °C: 10 mins	[66]
*ACT*	ACT-512F/ACT-783R	9 °C: 4 mins, (94 °C: 40 s, 52 °C: 30 s, 72 °C: 60 s) × 35 cycles, 72 °C: 10 mins	[67]
*GAPDH*	GDF1/GDR1	95 °C: 4 mins, (95 °C: 30 s, 60 °C: 30 s, 72 °C: 45 s) × 30 cycles, 72 °C: 7 mins	[68]
*TUB2*	Bt2b/T1	94 °C: 4 mins, (94 °C: 40 s, 52 °C: 30 s, 72 °C: 60 s) × 35 cycles, 72 °C: 10 mins	[69]

### Phylogenetic analysis

The resulting sequences were used to query GenBank via BLAST (http://blast.ddbj.nig.ac.jp/top-e.html). Sequences obtained in this study and from previous works, along with entries from the GenBank database, were used for phylogenetic analysis (Table 2). Multiple sequence alignment for each locus was performed using MUSCLE [22] with manual adjustments in BioEdit v6.0.7. The aligned sequences of the four loci, including the internal transcribed spacer (ITS) of ribosomal DNA, glyceraldehyde-3-phosphate dehydrogenase (*GAPDH*), actin (*ACT*), and beta-tubulin (*TUB2*) genes were concatenated into a single dataset using BioEdit v6.0.7 for phylogenetic analysis. Maximum likelihood (ML) phylogenetic trees were constructed using RAxML-HPC2 v8.2.12 on the CIPRES Science Gateway with the GTRCAT model and 1000 bootstrap replicates for statistical support [23]. Phylogenetic trees were visualized using TreeView v32 and edited in Adobe Illustrator v25.2.3.

**Table 2:** Sequences of *Colletotrichum* spp. used in the phylogenetic analysis. Species, strains, and GenBank accession numbers generated in this study are presented in bold.

Species	Strain/ isolate	Accession Number			
		ITS	*ACT*	*GAPDH*	*TUB2*
**Orchidearum complex**					
***C. orchidearum***	**SDBR-CMUPH1**	**PV478438**	**PV548285**	**PV548306**	**PV548295**
	CBS 135131T	MG600738	MG600944	MG600800	MG601005
	CBS 136877	MG600739	MG600945	MG600801	MG601006
*C. sojae*	ATCC 62257T	MG600749	MG600954	MG600810	MG601016
	CBS 128510	MG600751	MG600956	MG600812	MG601018
*C. cattleyicola*	CBS 170.49T	MG600758	MG600963	MG600819	MG601025
*C. syngoniicola*	LC8894T	MZ595863	MZ664161	MZ664117	MZ673982
	LC8895	MZ595864	MZ664162	MZ664118	MZ673983
*C. piperis*	IMI 7139T	MG600760	MG600964	MG600820	MG601027
*C. musicola*	CBS 132885T	MG600736	MG600942	MG600798	MG601003
*C. vittalense*	CBS 181.82T	MG600734	MG600940	MG600796	MG601001
*C. reniforme*	LC8230T	MZ595847	MZ664145	MZ664110	MZ673968
	LC8248	MZ595850	MZ664148	MZ664111	MZ673971
*C. plurivorum*	CBS125474T	MG600718	MG600925	MG600781	MG600985
	LC8337	MZ595855	MZ664153	MZ664115	MZ673976
**Magnum complex**					
***C. okinawense***	**SDBR-CMUPY1**	**PV478439**	**PV548286**	**PV548307**	**PV548296**
	MAFF 240517T	MG600767	MG600971	MG600827	MG601034
	PP3	MK649935	MK790071	MK790069	MK790073
*C. brevisporum*	BCC 38876T	JN050238	JN050216	JN050227	JN050244
	CBS 129958	MG600763	MG600967	MG600823	MG601030
*C. lobatum*	IMI 79736T	MG600768	MG600972	MG600828	MG601035
*C. merremiae*	CBS 124955T	MG600765	MG600969	MG600825	MG601032
*C. magnum*	CBS 519.97T	MG600769	MG600973	MG600829	MG601036
*C. magnum*	IMI 391662	MG600771	MG600975	MG600831	MG601038
*C. cacao*	CBS 119297T	MG600772	MG600976	MG600832	MG601039
**Acutatum complex**					
***C. nymphaeae***	**SDBR-CMUSB1**	**PV478440**	**PV548287**	**PV548308**	**PV548297**
	CBS 515.78T	JQ948197	JQ949518	JQ948527	JQ949848
	MAFF242590	MK628988	MK680658	MK645705	MK680724
*C. scovillei*	CBS 126529	JQ948267	JQ949588	JQ948597	JQ949918
*C. guajavae*	IMI 350839T	JQ948270	JQ949591	JQ948600	JQ949921
*C. eriobotryae*	GLMC 1835T	MF772487	MN191648	MF795423	MF795428
*C. paxtonii*	IMI 165753T	JQ948285	JQ949606	JQ948615	JQ949936
*C. simmondsii*	CBS 122122T	JQ948276	JQ949597	JQ948606	JQ949927
*C. melonis*	CBS 159.84T	JQ948194	JQ949515	JQ948524	JQ949845
*C. tamarilloi*	CBS 114.14T	JQ948184	JQ949505	JQ948514	JQ949835
*C. fioriniae*	CBS 128517T	JQ948292	JQ949613	JQ948622	JQ949943
*C. acutatum*	CBS 112996T	JQ005776	JQ005839	JQ948677	JQ005860
	CBS 979.69	JQ948400	JQ949721	JQ948731	JQ950051
*C. arboricola*	CBS 144795T	MH817944	MH817956	MH817950	MH817962
*C. salicis*	CBS 607.94T	JQ948460	JQ949781	JQ948791	JQ950111
*C. schimae*	NN046984T	MZ595885	MZ664183	MZ664105	MZ674003
*C. citri*	CBS134233T	KC293581	KY855973	KC293741	KC293661
*C. wanningense*	CGMCC 3.18936T	MG830462	MG830270	MG830318	MG830286
**Boninense complex**					
***C. brasiliense***	**SDBR-CMUPF1**	**PV478437**	**PV548284**	**PV548305**	**–**
	CBS 128501T	JQ005235	JQ005583	JQ005322	JQ005669
	CBS 128528	JQ005234	JQ005582	JQ005321	JQ005668
*C. hippeastri*	CBS 125376T	JQ005231	JQ005579	JQ005318	JQ005665
*C. parsonsiae*	CBS 128525T	JQ005233	JQ005581	JQ005320	JQ005667
*C. condaoense*	CBS 134299T	MH229914	–	MH229920	MH229923
*C. phyllanthi*	CBS 175.67T	JQ005221	JQ005569	JQ005308	JQ005655
*C. camelliae-japonicae*	CGMCC3.18118T	KX853165	KX893576	KX893584	KX893580
*C. citricola*	CBS 134228T	KC293576	KC293616	KC293736	KC293656
*C. annellatum*	CBS 129826T	JQ005222	JQ005570	JQ005309	JQ005656
*C. karstii*	CORCG6T	HM585409	HM581995	HM585391	HM585428
*C. constrictum*	CBS 128504T	JQ005238	JQ005586	JQ005325	JQ005672
*C. dacrycarpi*	CBS 130241T	JQ005236	JQ005584	JQ005323	JQ005670
*C. catinaense*	CBS 142417T	KY856400	KY855971	KY856224	KY856482
*C. novae-zelandiae*	CBS 128505T	JQ005228	JQ005576	JQ005315	JQ005662
*C. limonicola*	CBS 142410T	KY856472	KY856045	KY856296	KY856554
*C. feijoicola*	CBS 144633T	MK876413	MK876466	MK876475	MK876507
*C. petchii*	CBS 378.94T	JQ005223	JQ005571	JQ005310	JQ005657
*C. chamaedoreae*	NN052885T	MZ595890	MZ664188	MZ664084	MZ674008
*C. brassicicola*	CBS 101059T	JQ005172	JQ005520	JQ005259	JQ005606
*C. beeveri*	CBS 128527T	JQ005171	JQ005519	JQ005258	JQ005605
*C. cymbidiicola*	IMI 347923T	JQ005166	JQ005514	JQ005253	JQ005600
*C. torulosum*	CBS 128544T	JQ005164	JQ005512	JQ005251	JQ005598
*C. boninense*	CBS 123755T	JQ005153	JQ005501	JQ005240	JQ005588
**Truncatum complex**					
***C. truncatum***	**SDBR-CMUCHL1**	**PV478433**	**PV548280**	**PV548301**	**PV548291**
	CBS 151.35T	GU227862	GU227960	GU228254	GU228156
	CTM37	JX971160	JX975392	KC109615	KC109495
*C. curcumae*	IMI 288937T	GU227893	GU227991	GU228285	GU228187
*C. acidae*	MFLUCC 17.2659T	MG996505	MH003697	MH003691	MH003700
	MFLU 18–0233	MG996506	MH003698	MH003692	MH003701
*C. subacidae*	LC13857T	MZ595846	MZ664144	MZ664068	MZ673967
	MH0566	MZ595875	MZ664173	MZ664070	MZ673994
*C. fusiforme*	MFLU 13.0291T	KT290266	KT290251	KT290255	KT290256
**Gloeosporioides complex**					
***C. fructicola***	**SDBR-CMUADH1**	**PV478431**	**PV548278**	**PV548299**	**PV548289**
	ICMP 18581T	JX010165	JX009501	JX010033	JX010405
	ICMP 18613	JX010167	JX009491	JX009998	JX010388
*C. chrysophilum*	CMM4268T	KX094252	KX093982	KX094183	KX094285
*C. nupharicola*	ICMP 18187T	JX010187	JX009437	JX009972	JX010398
*C. alienum*	ICMP 12071T	JX010251	JX009572	JX010028	JX010411
*C. hystricis*	CBS 142411T	KY856450	KY856023	KY856274	KY856532
*C. viniferum*	CBS 130643T	JN412804	JN412795	JN412798	–
***C. musae***	**SDBR-CMUBN1**	**PV478432**	**PV548279**	**PV548300**	**PV548290**
	CBS 116870T	HQ596292	–	HQ596299	HQ596280
	CBS 125356	KC566800	KC566946	KC566654	KC566222
*C. hebeiense*	MFLUCC 13-0726T	KF156863	KF377532	KF377495	KF288975
*C. conoides*	CGMCC 3.17615T	KP890168	KP890144	KP890162	KP890174
*C. aenigma*	ICMP18608T	JX010244	JX009443	JX010044	JX010389
***C. asianum***	**SDBR-CMUMGK1**	**PV478434**	**PV548281**	**PV548302**	**PV548292**
	**SDBR-CMUMGN1**	**PV478436**	**PV548283**	**PV548304**	**PV548294**
	ICMP 18580T	JX010196	JX009584	JX010053	JX010406
	CMM4057	KC329792	KC533747	KC517168	KC517278
*C. tainanense*	CBS 143666T	MH728818	MH781475	MH728823	MH846558
*C. salsolae*	ICMP 19051T	JX010242	JX009562	JX009916	JX010403
*C. artocarpicola*	MFLUCC 18-1167T	MN415991	MN435570	MN435568	MN435567
*C. endophyticum*	MFLUCC 13-0418T	KC633854	KF306258	KC832854	MZ673954
*C. proteae*	CBS 132882T	KC297079	KC296940	KC297009	KC297101
*C. rhizophorae*	MFLUCC17-1927T	OR828933	OR840847	OR840870	OR840864
	MFLUCC17–1911	OR828934	OR840848	OR840871	OR840865
*C. tropicale*	ICMP 18653T	JX010264	JX009489	JX010007	JX010407
*C. aeschynomenes*	ICMP 17673T	JX010176	JX009483	JX009930	JX010392
*C. makassarense*	CBS 143664T	MH728812	MH781480	MH728820	MH846563
*C. gloeosporioides*	ICMP 17821T	JX010152	JX009531	JX010056	JX010445
	JZB330251	MZ822125	MZ826261	MZ826181	MZ826241
*C. temperatum*	CBS133122T	JX145159	MZ664125	MZ664045	JX145211
*C. chiangmaiense*	MFLUCC 18-0945T	MW346499	MW655578	MW548592	–
*C. camelliae*	CGMCC 3.14925T	KJ955081	KJ954363	KJ954782	KJ955230
*C. arecicola*	CGMCC 3.19667T	MK914635	MK935374	MK935455	MK935498
*C. henanense*	CGMCC 3.17354T	KJ955109	KM023257	KJ954810	KJ955257
*C. syzygiicola*	MFLUCC 10-0624T	KF242094	KF157801	KF242156	KF254880
*C. siamense*	ICMP 18578T	FJ972613	FJ907423	FJ972575	FJ907438
	HSI-3	OM654563	OM831342	OM831360	OM831384
***C. pandanicola***	**SDBR-CMUMGL1**	**PV478435**	**PV548282**	**PV548303**	**PV548293**
	**SDBR-CMUAD1**	**PV478430**	**PV548277**	**PV548298**	**PV548288**
	MFLUCC17-0571T	MG646967	MG646938	MG646934	MG646926
	SAUCC200204	MW786641	MW883694	MW846239	MW888969

### Preparation of medicinal plant extracts

The extraction method using ethanol was modified from the procedure described in Al-Otibi et al. [24]. Ethanol is widely used as a plant extraction solvent because it effectively solubilizes a broad range of bioactive constituents, including flavonoids, phenolics, and other antimicrobial metabolites [24,25]. Ten different medicinal plants, using various parts, were collected: lemongrass (*Cymbopogon citratus*), clove (*Syzygium aromaticum*), sea holly (*Acanthus ebracteatus*), heart-leaved moonseed (*Tinospora cordifolia*), turmeric (*Curcuma longa*), black pepper (*Piper nigrum*), cassumunar ginger (*Zingiber montanum*), Indian gooseberry (*Phyllanthus emblica*), cinnamon (*Cinnamomum verum*), and kariyat (*Andrographis paniculata*). The plant materials were dried at 50°C and ground into a fine powder. Briefly, 100 g of dried plant powder was soaked in approximately 500 mL of absolute ethanol (1:5 w/v) and incubated for 72 hours. The mixture was then filtered using Whatman No.1 filter paper. After filtration, the extracts were evaporated using a rotary evaporator (Buchi Rota vapor, Switzerland) at a temperature below 50°C and a rotation speed of 90 rpm to obtain semi-solid products. The extracts were then weighed to determine the percentage extraction yield using the equation from Akwongo et al. [26]: Extract yield (%) = (weight of dried extract/weight of dried plant sample) × 100

The crude extracts were dissolved in absolute ethanol and stored in a refrigerator for further use in antifungal tests.

The crude extracts obtained from the respective plants were dissolved in absolute ethanol to achieve a final concentration of 100 mg/mL. The potential antifungal activity of selected crude plant extracts was evaluated using the minimum inhibitory concentration (MIC) method, with concentrations adjusted to 100, 50, 25, 12.5, 6.25, 3.125, 1.5625, and 0.78125 mg/ml. Pure methyl eugenol (Sigma Aldrich, USA) was diluted two-fold with ethanol to a final concentration of 250 mg/mL for comparison with the plant crude extracts in antifungal activity tests. Methyl eugenol, a compound with known antifungal properties that is commonly extracted from various plant species, was selected as a positive control for comparison with the plant extracts. However, methyl eugenol can exhibit lower antifungal efficacy, as methylation of the phenolic hydroxyl group has been reported to reduce antifungal activity [27]. Consequently, methyl eugenol was applied at a higher concentration than the plant extracts.

### Evaluation of antifungal activity of plant extracts against *Colletotrichum* species

The paper disc diffusion method was used to screen the antifungal activity of the plant extracts on PDA. Fungal spores were collected from the spore masses of each isolate and suspended in sterile distilled water to achieve a concentration of approximately 1.0 × 10^7^ spores/mL. The spore suspension was evenly spread over the surface of agar plates using a sterile cotton swab. Sterile filter paper discs (6 mm diameter) were impregnated with crude extract in absolute ethanol, air-dried at room temperature (25 °C) under aseptic conditions in a laminar flow hood, and placed on the agar surface. Antifungal activity was assessed after 24–48 hours of incubation at 28°C. The diameters (cm) of the inhibition zones were measured, and antifungal activity was expressed as the mean inhibition zone based on three replicates per treatment.

### GC-MS analysis

GC-MS analysis was performed using an Agilent 7890A gas chromatograph coupled with an Agilent 5975C mass spectrometer. The column used was an HP-5MS UI capillary column (30 m in length × 250 μm in diameter × 0.25 μm in thickness). The oven temperature was programmed to increase from 40°C to 200°C at a rate of 6°C/min, then from 200°C to 280°C at 30°C/min, and was finally held at 300°C for 10 minutes. A post-run was conducted at 280°C for 10 minutes. Helium was used as the carrier gas at a constant flow rate of 1 mL/min. The injector and detector temperatures were both set to 250°C. GC-MS analysis was carried out by injecting 1 μL of the sample (0.1% in absolute methanol) in splitless mode, using scan mode detection. Quinaldine was used as an external standard. The chemical constituents of the clove extract were identified by comparing retention times and mass spectra with those in the Wiley mass spectral library (W8N08, John Wiley & Sons, Inc., USA).

### Phytochemical constituents by LC-MS analysis of selected plant extract

Plant extracts exhibiting antifungal activity were selected for this experiment. High-performance liquid chromatography (HPLC) separation was performed using a Poroshell 120 EC-C18 column (100 mm × 2.1 mm, 2.7 μm, Agilent Company, USA), maintained at 50°C. A 10 μl aliquot of crude plant extract, prepared in 70% methanol containing 25 ng/ml sulfadimethoxine, was injected for analysis. The mobile phase consisted of solvent A (deionized water with 0.1% formic acid, *v*/*v*) and solvent B (acetonitrile with 0.1% formic acid, *v*/*v*). An exploratory gradient elution was applied as follows: 55–75% solvent B from 10.5 to 12.5 minutes, followed by 100% solvent B from 14.0 to 17.0 minutes, at a constant flow rate of 0.4 mL/min. Samples were analyzed in all-ion MS/MS acquisition modes, with a collision energy of 20 eV in positive ion mode and 10 eV in negative ion mode.

Mass spectrometry analysis was performed using an LC-QTOF 6545XT system (Agilent Technologies, USA) equipped with an electrospray ionization (ESI) source utilizing Jet Stream technology. The instrument was operated under the following conditions: drying gas (N_2_) flow rate, 13 L/min; drying gas temperature, 325°C; nebulizer pressure, 45 psi; sheath gas temperature, 275°C; sheath gas flow, 12 L/min; and capillary voltage, 4000 V (positive mode) or 3000 V (negative mode). Each sample was analyzed in both positive and negative ionization modes over an *m/z* range of 40–1700 for MS1 and 25–1000 for MS2 acquisitions. Data processing was performed using MS-DIAL version 5.3, utilizing ESI (±) MS/MS data. Compound annotation was conducted using authentic standards and referenced against the Fiehn/Vaniya Natural Product Database and the BMDMS-NP library [28].

## Results

### Isolation and morphological characterization of *Colletotrichum* species from anthracnose samples

**Fig 1:**
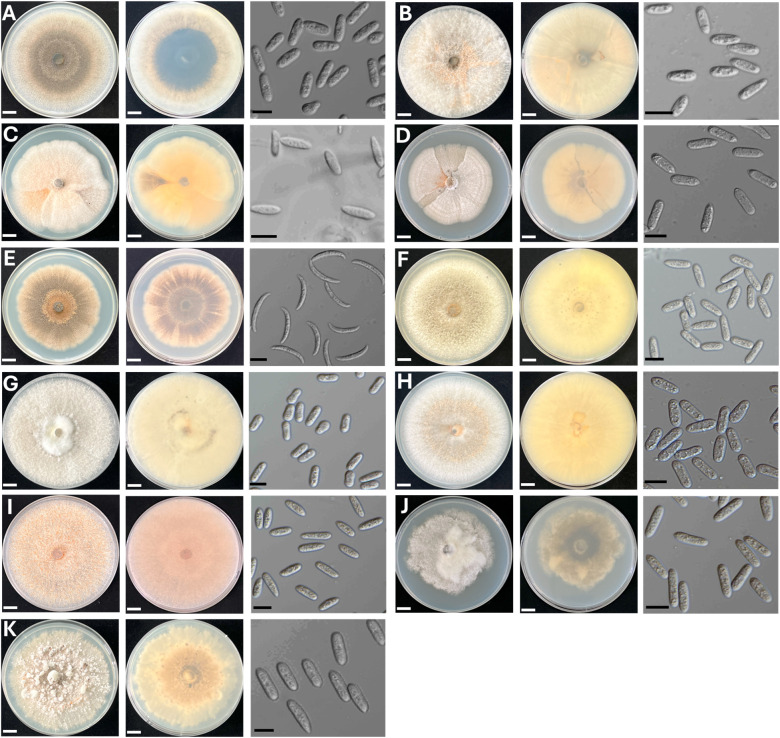
Morphological characteristics of 11 *Colletotrichum* isolates causing anthracnose on fruits and ornamental plants in Thailand. *Colletotrichum orchidearum* PH1 **(A)**, *C. okinawense* PY1 **(B)**, *C. nymphaeae* SB1 **(C)**, *C. brasiliense* PF1 **(D)**, *C. truncatum* CHL1 **(E)**, *C. fructicola* ADH1 **(F)**, *C. pandanicola* MGL1 **(G)**, *C. pandanicola* AD1 **(H)**, *C. musae* BN1 **(I)**, *C. asianum* MGK1 **(J)**, and *C. asianum* MGN1 **(K)**. From left to right, each panel shows the upper surface of colonies grown on potato dextrose agar (PDA) at 14 days after inoculation, the reverse side of the colonies, and the conidia or ascospores. Scale bars: 1 cm for culture plate images; 10 μm for spore micrographs.

In total, eleven *Colletotrichum* isolates were obtained from eleven diseased samples: two from mango fruits (cv. “Num Dok Mai” and “Kaew Khamin”), one from a mango leaf, one from a strawberry, one from a passion fruit, one from a papaya, one from a banana, one from a chili, two from avocados (cv. “Hass” and “Buccaneer”), and one from an ornamental philodendron. All isolates were cultivated on PDA. Most *Colletotrichum* isolates exhibited white to grey aerial mycelia, while some displayed yellow to pale brown mycelia (Fig 1, Table 3). Orange spore masses were visible on the aerial mycelia of some isolates. On the reverse side, nearly all fungal colonies were pale yellow to yellowish gray, while a few showed grayish olive to olive black coloration at the center. None of the isolates produced pigmentation in the PDA medium. The conidia of nearly all isolates were cylindrical or oval with varying lengths and widths, except for isolate CHL1, which exhibited curved or sickle-shaped conidia. All *Colletotrichum* isolates were identified through multi-gene molecular phylogenetic analyses.

**Table 3:** Morphological characterization of *Colletotrichum* isolated from fruits and ornamental plants.

Isolate	Isolation sources	Colony characteristics	Conidia			Identified as
			Length (μm)	Width (μm)	Shape	
PH1	Philodendron	White to grey to brown, less fluffy mycelia with orange visible conidial masses; reverse brownish black in center	14.24 ± 1.56 (10.64–18.15)	4.97 ± 0.36 (4.29–5.83)	Ovoid-to-cylindrical	*C. orchidearum*
PY1	Papaya	White to gray, fluffy mycelia with orange visible conidial masses; reverse yellowish gray	11.36 ± 0.91 (8.23–13.11)	5.03 ± 0.52 (3.94–5.96)	Cylindrical with rounded end	*C. okinawense*
SB1	Strawberry	White cottony aerial mycelia with orange visible conidial masses; reverse pale yellow to light orange	13.475 ± 2.10 (9.35–17.56)	3.65 ± 0.37 (2.57–4.52)	Cylindrical	*C. nymphaeae*
PF1	Passion fruit	White to grey, less fluffy mycelia with orange visible conidial masses in center; reverse pale yellow	14.43 ± 1.17 (12.61–18.41)	4.79 ± 0.35 (4.09–5.48)	Cylindrical	*C. brasiliense*
CHL1	Chili	Yellow to brown, floccose mycelia; reverse pale yellow with some areas of dark olive brown	24.16 ± 1.99 (17.91–29.02)	2.99 ± 0.32 (2.25–3.85)	Curved or sickle-shaped	*C. truncatum*
ADH1	Hass avocado	White to grey cottony, fluffy mycelia; reverse pale yellow	13.49 ± 1.26 (10.79–16.94)	4.28 ± 0.47 (3.35–5.49)	Cylindrical	*C. fructicola*
MGL1	Mango leaf	White to grey cottony, fluffy mycelia; reverse pale yellow	12.50 ± 0.93 (9.46–15.15)	3.59 ± 0.41 (2.88–4.53)	Oval with blunt ended	*C. pandanicola*
AD1	Buccaneer avocado	White to gray, fluffy mycelia with orange visible conidial masses; reverse pale yellow	14.91 ± 0.95 (12.34–16.86)	5.25 ± 0.43 (4.22–6.44)	Oval with blunt ended	*C. pandanicola*
BN1	Banana	Yellowish white less fluffy aerial mycelia with orange visible conidial masses; reverse light orange	12.48 ± 1.31 (9.35–16.09)	5.06 ± 0.36 (4.35–5.93)	Ellipsoid	*C. musae*
MGK1	Mango	White to off white, with dense whitish mycelia; reverse dark grayish olive to olive black in center	15.78 ± 1.50 (12.24–19.39)	4.39 ± 0.43 (3.29–5.49)	Cylindrical	*C. asianum*
MGN1	Mango	Off-white to yellowish white, floccose mycelia with orange visible conidial masses; reverse pale yellow to dark grayish yellow in center	14.99 ± 1.78 (10.82–18.24)	4.79 ± 0.44 (3.25–5.79)	Cylindrical	*C. asianum*

### Multi-gene molecular phylogenetic analyses

**Fig 2:**
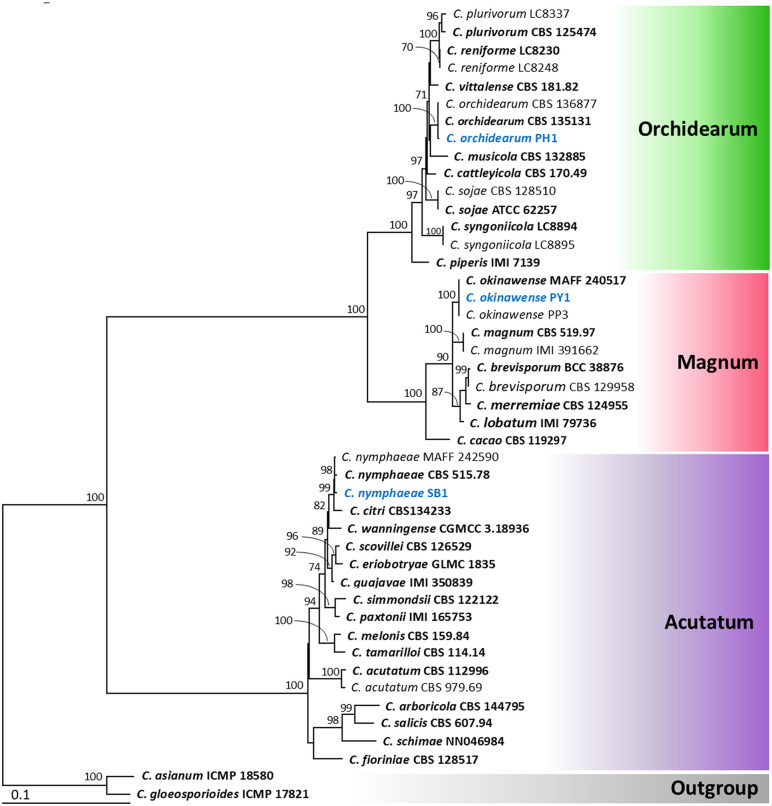
Maximum likelihood tree based on analysis of the combined dataset of ITS region, *GAPDH*, *ACT*, and *TUB2* partial genes for the *C. orchidearum*, *C. magnum*, and *C. acutatum* complexes. Strains isolated in this study are shown in blue. The bar indicates substitution per site. *Colletotrichum asianum* strain ICMP 18580 and *C. gloeosporioides* strain ICMP 17821 were used as outgroups. Type strains are shown in bold.

**Fig 3:**
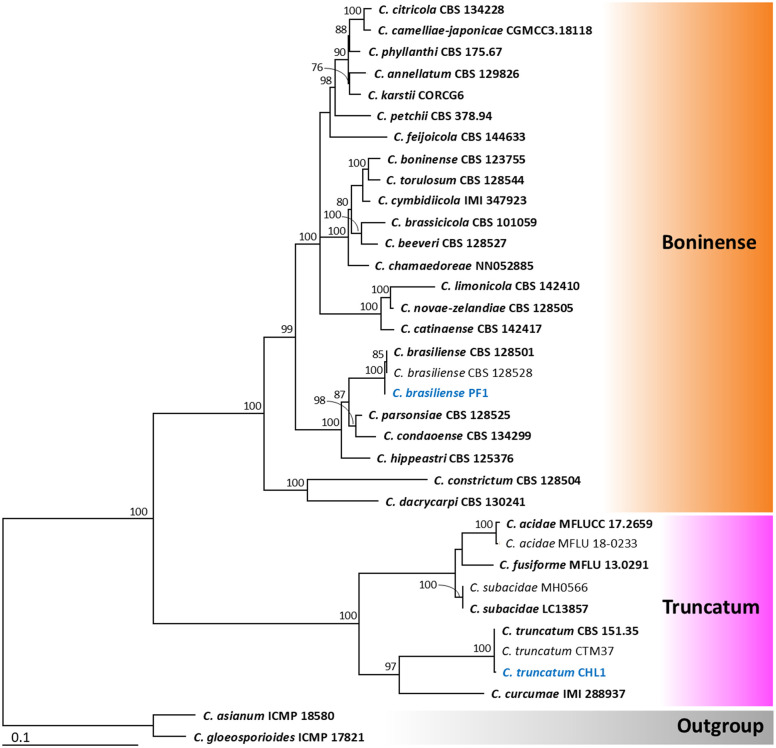
Maximum likelihood tree based on analysis of the combined dataset of ITS region, *GAPDH*, *ACT*, and *TUB2* partial genes for the *C. boninense* and *C. truncatum* complexes. Strains isolated in this study are shown in blue. The bar indicates substitution per site. *Colletotrichum asianum* strain ICMP 18580 and *C. gloeosporioides* strain ICMP 17821 were used as outgroups. Type strains are shown in bold.

**Fig 4:**
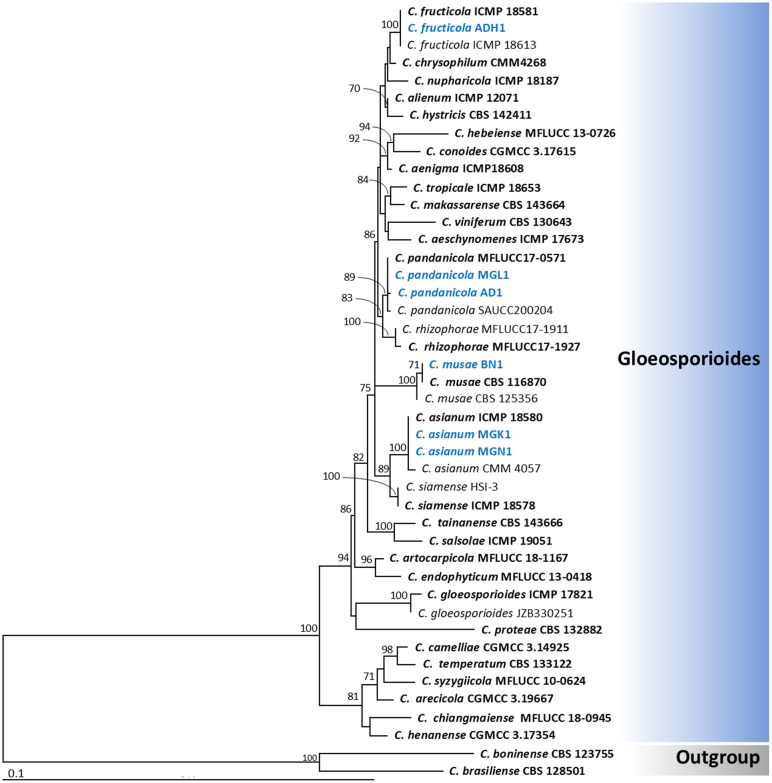
Maximum likelihood tree based on analysis of the combined dataset of ITS region, *GAPDH*, *ACT*, and *TUB2* partial genes for the *C. gloeosporioides* complex. Strains isolated in this study are shown in blue. The bar indicates substitution per site. *Colletotrichum boninense* strain CBS 123755 and *C. brasilliense* strain CBS 128501 were used as outgroups. Type strains are shown in bold.

Based on the maximum likelihood (ML) tree, the dataset comprising four genes (ITS, *ACT*, *TUB2*, and *GAPDH*) was divided into six *Colletotrichum* species complexes: *Orchidearum*, *Magnum*, *Acutatum*, *Boninense*, *Truncatum*, and *Gloeosporioides* clades, in accordance with the percentage identity results of the *ACT*, *TUB2*, and *GAPDH* genes. Phylogenetic trees obtained from the ML analysis are shown in Figs 2 to 4. The results revealed that isolates PH1, PY1, SB1, PF1, and CHL1 were identified as *Colletotrichum orchidearum* (*Orchidearum* clade), *C. okinawense* (*Magnum* clade), *C. nymphaeae* (*Acutatum* clade), *C. brasiliense* (*Boninense* clade), and *C. truncatum* (*Truncatum* clade), respectively (Fig 2 and 3). Six additional isolates were placed within the *Gloeosporioides* clades (Fig 4). Among these, isolates ADH1 and BN1 were identified as *C. fructicola* and *C. musae*, respectively. Both AD1 and MGL1 were identified as *C. pandanicola*, while MGK1 and MGN1 were identified as *C. asianum*. All isolates clustered with the type strains of their respective species. Therefore, the eleven *Colletotrichum* isolates obtained in this study were identified as belonging to nine different species, based on both morphological characteristics and multi-gene molecular phylogenetic analyses. All fungal isolates were deposited in the SDBR Laboratory’s Culture Collection under numbers SDBR-CMUPH1, SDBR-CMUPY1, SDBR-CMUSB1, SDBR-CMUPF1, SDBR-CMUCHL1, SDBR-CMUADH1, SDBR-CMUMGL1, SDBR-CMUAD1, SDBR-CMUBN1, SDBR-CMUMGK1, and SDBR-CMUMGN1.

### Yield of plant crude extracts

The extraction yields of the ten medicinal plants varied, with the percentage of ethanolic extracts ranging from 0.711% to 23.281% (Table 4). The highest yield was obtained from cinnamon, while the lowest was from sea holly. The second to fifth highest extraction yields were from Indian gooseberry (7.44%), turmeric (7.34%), cassumunar ginger (5.40%), and clove (5.29%), respectively.

**Table 4:** Percentage yield of ethanolic extracts from ten medicinal plants in Thailand.

Scientific name	Family	Common name	Plant parts used	% yield
*Cymbopogon citratus*	*Poaceae*	Lemongrass	Leaves	1.79
*Syzygium aromaticum*	*Myrtaceae*	Clove	Flower	5.29
*Acanthus ebracteatus*	*Acanthaceae*	Sea holly	Leaves	0.71
*Tinospora cordifolia*	*Menispermaceae*	Heart-leaved moonseed	Stem	1.16
*Curcuma longa*	*Zingiberaceae*	Turmeric	Rhizomes	7.34
*Piper nigrum*	*Piperaceae*	Black pepper	Seed	4.51
*Zingiber montanum*	*Zingiberaceae*	Cassumunar ginger	Rhizome	5.40
*Phyllanthus emblica*	*Euphorbiaceae*	Indian gooseberry	Fruit	7.44
*Cinnamomum verum*	*Lauraceae*	Cinnamon	Inner bark	23.28
*Andrographis paniculata*	*Acanthaceae*	Kariyat	Leaves	3.84

### Evaluation of antifungal activity of plant extracts against *Colletotrichum* species

**Fig 5:**
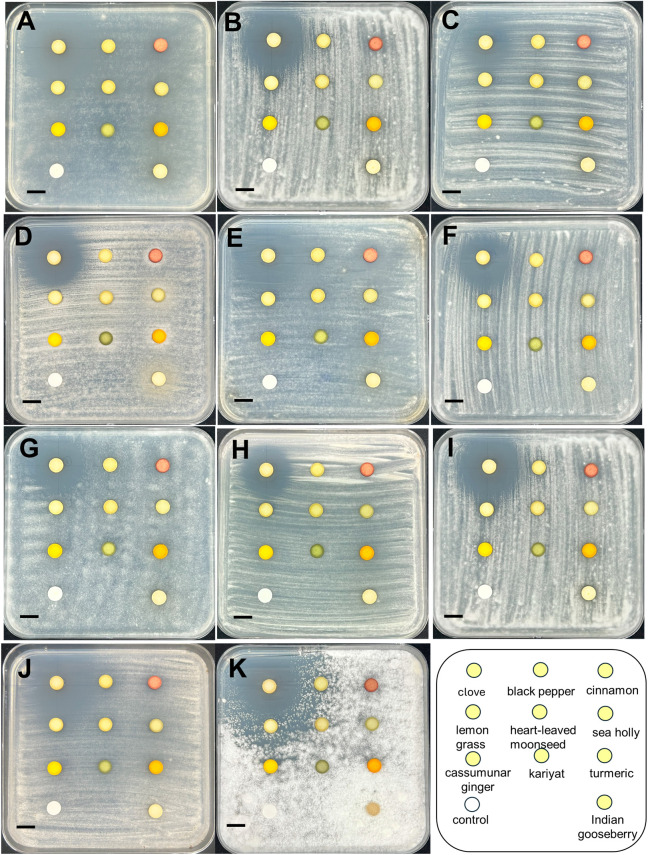
Primary screening of ethanolic extracts from ten medicinal plants at a concentration of 100 mg/mL, against all isolated *Colletotrichum* species. *Colletotrichum orchidearum* PH1 **(A)**, *C. okinawense* PY1 **(B)**, *C. nymphaeae* SB1 **(C)**, *C. brasiliense* PF1 **(D)**, *C. truncatum* CHL1 **(E)**, *C. fructicola* ADH1 **(F)**, *C. pandanicola* MGL1 **(G)**, *C. pandanicola* AD1 **(H)**, *C. musae* BN1 **(I)**, *C. asianum* MGK1 **(J)**, and *C. asianum* MGN1 **(K)**. Scale bars = 1 cm.

The effect of various ethanol-based plant extracts on *Colletotrichum* growth was evaluated using the paper disc diffusion method. Fungi were cultured on PDA in the absence (control) or presence (treatment) of each plant extract. At a concentration of 100 mg/mL, the clove ethanolic extract strongly inhibited mycelial growth in all *Colletotrichum* isolates, producing inhibition zones ranging from 2.70 to 4.50 cm (Fig 5). Among the 10 plant extracts tested, only the clove extract demonstrated significant antifungal activity and was therefore selected for further MIC analysis.

### Determination of minimum inhibitory concentrations (MICs) of selected plant extract

**Fig 6:**
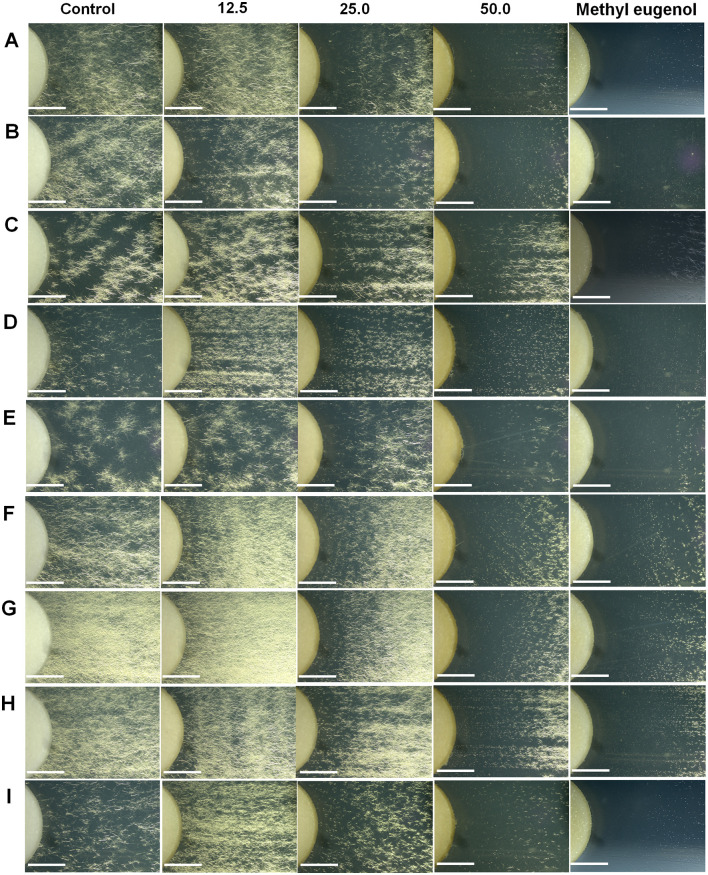
Inhibition zones of mycelium growth of all isolated *Colletotrichum* species in the MIC test using ethanolic clove extract at concentrations of 50, 25, 12.5 mg/mL, compared with the ethanol (control) and methyl eugenol at 250 mg/mL. *Colletotrichum orchidearum* PH1 **(A)**, *C. okinawense* PY1 **(B)**, *C. nymphaeae* SB1 **(C)**, *C. brasiliense* PF1 **(D)**, *C. truncatum* CHL1 **(E)**, *C. fructicola* ADH1 **(F)**, *C. pandanicola* MGL1 **(G)**, *C. musae* BN1 **(H)**, and *C. asianum* MGK1 **(I)**. Scale bars = 1 cm.

MICs were determined for the plant extract that exhibited antifungal activity against nine different *Colletotrichum* species. The antifungal activities of the clove ethanolic extract are summarized in Table 5 as inhibition-zone diameters. For most isolates, the MIC of the clove extract was 25 mg/mL; however, *C. orchidearum* and *C. okinawense* showed MIC values of 12.5 mg/mL (Fig 6). For comparison, purified methyl eugenol (50% v/v in ethanol) also inhibited the growth of all isolates, producing inhibition zones of varying diameters. The largest zones produced by methyl eugenol were observed for *C. okinawense* (4.03 cm), followed by *C. orchidearum* (3.80 cm). Microscopic examination at the same magnification revealed that the inhibition zone produced by eugenol completely prevented mycelial growth in *C. okinawense* and *C. orchidearum* (Fig 6A and I), in contrast to other *Colletotrichum* species.

**Table 5:** MIC of the ethanolic clove extract against all tested nine different *Colletotrichum* species.

*Colletotrichum* isolate	Inhibition zone (cm)				
	6.25 mg/mL	12.5 mg/mL	25 mg/mL	50 mg/mL	Eugenol (50% v/v)
*C. orchidearum* PH1	0.00 ± 0.00	1.08 ± 0.04A	1.84 ± 0.06A	2.83 ± 0.08B	3.80 ± 0.07B
*C. okinawense* PY1	0.00 ± 0.00	1.09 ± 0.01A	1.81 ± 0.05A	2.97 ± 0.06A	4.03 ± 0.11A
*C. nymphaeae* SB1	0.00 ± 0.00	0.00 ± 0.00B	1.50 ± 0.05B	2.32 ± 0.06EF	2.78 ± 0.11D
*C. brasiliense* PF1	0.00 ± 0.00	0.00 ± 0.00B	1.16 ± 0.06D	2.48 ± 0.06D	3.20 ± 0.14C
*C. truncatum* CHL1	0.00 ± 0.00	0.00 ± 0.00B	1.19 ± 0.11D	2.23 ± 0.03F	2.83 ± 0.04D
*C. fructicola* ADH1	0.00 ± 0.00	0.00 ± 0.00B	1.38 ± 0.09C	2.42 ± 0.13DE	2.78 ± 0.04D
*C. pandanicola* MGL1	0.00 ± 0.00	0.00 ± 0.00B	1.28 ± 0.18D	2.59 ± 0.07C	2.66 ± 0.05D
*C. musae* BN1	0.00 ± 0.00	0.00 ± 0.00B	1.37 ± 0.10C	2.23 ± 0.04F	2.68 ± 0.04D
*C. asianum* MGK1	0.00 ± 0.00	0.00 ± 0.00B	1.47 ± 0.03B	2.88 ± 0.03AB	3.03 ± 0.11C
*p-value*	ns	<0.0001**	<0.0001**	<0.0001**	<0.0001**

### GC-MS analysis of bioactive compounds in selected plant extract

GC-MS analysis of the ethanolic clove extract revealed the presence of various bioactive chemical compounds (Table 6). The major constituent identified was 2-methoxy-4-prop-2-enylphenol (eugenol, 72.417%), followed by (1R,4E,9S)-4,11,11-trimethyl-8-methylenebicyclo[7.2.0]undec-4-ene (β-caryophyllene, 12.125%), (2-methoxy-4-prop-2-enylphenyl) acetate (eugenyl acetate, 8.121%), and (1E,4E,8E)-2,6,6,9-tetramethylcycloundeca-1,4,8-triene (α-humulene, 3.268%).

**Table 6:** Chemical composition of ethanolic crude extract obtained from clove using GC-MS analysis.

RT (min)	Compounds (IUPAC name)	Formula	Area (%)
9.01	2-methylheptan-2-yl acetate	C_10_H_20_O_2_	0.011
10.46	nonan-2-one	C_9_H_18_O	0.005
12.29	(3*E*)-4,8-dimethylnona-1,3,7-triene	C_11_H_18_	0.012
13.10	methyl 2-hydroxybenzoate	C_8_H_8_O_3_	0.024
13.95	cyclobutadiene	C_4_H_4_	0.002
14.37	(1*R*,5*S*,6*R*,*7S*,*10R*)-4,10-dimethyl-7-propan-2-yltricyclo[4.4.0.0^1,5^]dec-3-ene	C_15_H_24_	0.021
16.89	2-methoxy-4-prop-2-enylphenol	C_10_H_12_O_2_	72.417
17.22	1,3-dimethyl-8-propan-2 yltricyclo[4.4.0.0^2,7^]dec-3-ene	C_15_H_24_	0.355
17.39	2-methoxy-4-prop-2-enylphenol	C_10_H_12_O_2_	0.022
17.55	(1*S*,2*S*,4*R*)-1-ethenyl-1-methyl-2,4-bis(prop-1-en-2-yl) cyclohexane	C_15_H_24_	0.029
18.29	(1*R*,4*E*,9*S*)-4,11,11-trimethyl-8-methylidenebicyclo [7.2.0]undec-4-ene	C_15_H_24_	12.125
18.78	(1*E*,4*E*,8*E*)-2,6,6,9-tetramethylcycloundeca-1,4,8-triene	C_15_H_24_	3.268
19.11	(1*aR*,4*aS*,7*R*,7*aR*,7*bS*)-1,1,7-trimethyl-4-methylidene-2,3,4*a*,5,6,7,7*a*,7*b*-octahydro-1*aH*-cyclopropa[e]azulene	C_15_H_24_	0.019
19.24	(*1S*,8*aR*)-4,7-dimethyl-1-propan-2-yl-1,2,3,5,6,8*a*-hexahydronaphthalene	C_15_H_24_	0.047
19.31	(1*S*,4a*R*,8*aS*)-4,7-dimethyl-1-propan-2-yl-1,2,4*a*,5,6,8*a*-hexahydronaphthalene	C_15_H_24_	0.061
19.41	(1*S*,4*aS*,8a*R*)-7-methyl-4-methylidene-1-propan-2-yl-2,3,4*a*,5,6,8*a*-hexahydro-1*H*-naphthalene	C_15_H_24_	0.027
19.91	(1*S*,4*aR*,8*aS*)-4,7-dimethyl-1-propan-2-yl-1,2,4*a*,5,8,*8a*-hexahydronaphthalene	C_15_H_24_	0.079
20.19	(1*S*,4*aR*,8*aR*)-7-methyl-4-methylidene-1-propan-2-yl-2,3,4*a*,5,6,8*a*-hexahydro-1*H*-naphthalene	C_15_H_24_	0.023
20.41	(2-methoxy-4-prop-2-enylphenyl) acetate	C_12_H_14_O_3_	8.121
20.67	(1*S*,4*aR*,8*aR*)-4,7-dimethyl-1-(propan-2-yl)-1,2,4*a*,5,6,8*a*-hexahydronaphthalene	C_15_H_24_	0.043
20.85	(1*S*)-4,7-dimethyl-1-propan-2-yl-1,2-dihydronaphthalene	C_15_H_20_	0.079
21.19	(5*Z*,7*E*)-dodeca-1,5,7-triene	C_12_H_20_	0.011
21.32	(3*Z*)-4,8,11,11-tetramethylbicyclo[7.2.0]undec-3-en-5-ol	C_15_H_26_O	0.020
21.53	(1*R*,*4R*,6*R*,10*S*)-4,12,12-trimethyl-9-methylidene-5-oxatricyclo[8.2.0.0^4,6^]dodecane	C_15_H_24_O	0.398
21.83	(1*R*)-1,5,5,8-tetramethyl-12-oxabicyclo[9.1.0]dodeca-3,7-diene	C_15_H_24_O	0.047
22.41	1,4-dimethyl-7-propan-2-ylidene-2,3,4,5,6,8-hexahydro-1*H*-azulene	C_15_H_24_	0.011
22.87	(1*S*,9*R*)-10,10-dimethyl-2,6-dimethylidenebicyclo[7.2.0]undecan-5-ol	C_15_H_24_O	0.069
23.48	1-(2,3,4-trimethoxyphenyl)ethanone	C_11_H_14_O_4_	0.311
24.91	Benzyl benzoate	C_14_H_12_O_2_	0.013

### Phytochemical constituents identified by LC-MS analysis of selected plant extract

LC-MS analysis of the clove ethanolic extract (Table 7) revealed a diverse array of bioactive metabolites. The flavonoids detected included quercetin, kaempferol, hispidulin, isosakuranin, myricetin, and delphinidin, while the phenolic compounds comprised gallic acid and pyrogallol. The terpenoid profile was dominated by pygenic acid and ursolic acid. Additional metabolites identified included alkaloids, catechol, resveratrol, and coumaric acid.

**Table 7:** Chemical composition of ethanolic crude extract obtained from clove using LC-MS analysis.

Group	Compounds (IUPAC name)	Synonyms	RT (min)	Ion type	Molecular formular	Arbitrary unit (a.u.)
Flavonoids and flavonoid glycosides	2-(3,4-dihydroxyphenyl)-3,5,7-trihydroxy-4H-chromen-4-one	Quercetin	5.1	[M-H]-	C_15_H_10_O_7_	31,359,352.43
	3,5,7-trihydroxy-2-(4-hydroxyphenyl)-4H-chromen-4-one	Kaempferol	6.8	[M-H]-	C_15_H_10_O_6_	21,554,196.74
	3,5,7-trihydroxy-2-(4-hydroxy-3-methoxyphenyl)-4H-chromen-4-one	Isorhamnetin	7.9	[M-H]-	C_16_H_12_O_7_	13,229,585.23
	(2S)-5,7-dihydroxy-2-(4-hydroxyphenyl)-2,3-dihydro-4H-chromen-4-one	Naringenin	7.4	[M-H]-	C_15_H_12_O_5_	3,085,076.13
	5,7-dihydroxy-2-(4-hydroxyphenyl)-6-methoxy-4H-chromen-4-one	Hispidulin	9.7	[M-H]-	C_16_H_12_O_6_	22,636,863.12
	(2S)-5-hydroxy-2-(4-methoxyphenyl)-7-[(2S,3R,4S,5S,6R) −3,4,5-trihydroxy-6-(hydroxymethyl)oxan-2-yl]oxy-2,3-dihydrochromen-4-one	Isosakuranin	6.0	[M-H]-	C_22_H_24_O_10_	21,591,424.06
	5,7-dihydroxy-2-(3-hydroxy-4-methoxyphenyl)-4H-chromen-4-one	Diosmetin	8.0	[M-H]-	C_16_H_12_O_6_	9,389,580.27
	2-methoxy-4-[(E)-prop-1-enyl]phenol	Isoeugenitol	3.4	[M-H]-	C_11_H_10_O_4_	9,214,625.86
	5,7-dihydroxy-2-(4-hydroxyphenyl)-3-methoxychromen-4-one	Isokaempferide	9.8	[M+H]^+^	C_16_H_12_O_6_	9,076,343.70
	2-methoxy-4-[7-methoxy-3-methyl-5-[(E)-prop-1-enyl]-2,3-dihydro-1-benzofuran-2-yl]phenol	Dehydrodi-isoeugenol	12.2	[M+H]^+^	C_20_H_22_O_4_	8,837,953.04
	2-(7-methoxy-4-oxo-4H-chromen-3-yl)acetic acid	Anhydrobrazilic Acid	3.9	[M+H]^+^	C_12_H_10_O_5_	12,818,691.40
	3,5,7-trihydroxy-2-(3,4,5-trihydroxyphenyl)-4H-chromen-4-one	Myricetin	5.9	[M-H]^-^	C_15_H_10_O_8_	28,518,056.22
	3,5,7-trihydroxy-2-(3,4,5-trihydroxyphenyl)-1λ⁴-chromen-1-ylium	Delphinidin	6.0	[M-2H]^-^	C_15_H_11_O_7_	23,370,869.89
Phenolics	3,4,5-trihydroxybenzoic acid	Gallic acid	1.7	[M-H]^-^	C_7_H_6_O_5_	122,941,513.11
	(1S,3R,4S,5R)-1,3,4,5-tetrahydroxycyclohexane-1-carboxylic acid	D-quinic acid	0.7	[M-H]^-^	C_7_H_12_O_6_	25,070,556.05
	(1R,3R,4S,5R)-3-[(E)-3-(3,4-dihydroxyphenyl)prop-2-enoyl]oxy-1,4,5-trihydroxycyclohexane-1-carboxylic acid	Neochlorogenic acid	3.4	[M-H]^-^	C_16_H_18_O_9_	125,849,013.61
	6,7,13,14-tetrahydroxy-2,9-dioxatetracyclo[6.6.2.0⁴^,^¹⁶.0¹¹^,^¹⁵] hexadeca-1(15),4(16),5,7,11,13-hexaene-3,10-dione	Ellagic acid	5.2	[M-H]^-^	C_14_H_6_O_8_	24,842,622.09
	(2S,3R,4S,5R,6R)-5-hydroxy-6-(((3,4,5-trihydroxybenzoyl)oxy) methyl)tetrahydro-2H-pyran-2,3,4-triyl tris(3,4,5-trihydroxybenzoate)	1,2,3,6-tetra galloylglucose	4.9	[M-H]-	C_34_H_28_O_22_	21,946,120.61
	[3,5-dihydroxy-4,6-bis(3,4,5-trihydroxybenzoyloxy)oxan-2-yl]methyl 3,4,5-trihydroxybenzoate	1,3,6-tri-O-galloylglucose	3.7	[M-H]^-^	C_27_H_24_O_18_	15,293,993.98
	benzene-1,2,3-triol	Pyrogallol	1.7	[M-H]^-^	C_6_H_6_O_3_	160,393,749.63
	(E)-1-(4-Hydroxy-3-methoxyphenyl)dec-4-en-3-one	6-Shogaol	8.1	[M+H]^+^	C_17_H_24_O_3_	8,527,081.74
	(E)-3-(4-hydroxy-3-methoxyphenyl)prop-2-enal	Coniferaldehyde	12.6	[M+H]^+^	C_10_H_10_O_3_	9,343,344.61
	1-(2,4,5-trihydroxyphenyl)butan-1-one	2,4,5-Trihydroxy butyrophenone	7.0	[M-H]^-^	C_10_H_12_O_4_	32,853,619.54
	β-D-Glucopyranose 1,6-bis(3,4,5-trihydroxybenzoate)	1,6-bis-O-(3,4,5-trihydroxybenzoyl)- *β*-D-glucopyranose	0.7	[M-H]^-^	C_20_H_20_O_14_	30,070,842.12
Alkyl-phenylketones	1-(2-hydroxy-4,6-dimethoxy-3-methylphenyl)ethan-1-one	Methylxanthoxylin	10.3	[M+H]^+^	C_11_H_14_O_4_	8,732,644.42
Triterpenoids	(1S,2R,4aS,6aR,6aS,6bR,10S,11R,12aR,14bS)-10,11-dihydroxy-1,2,6a,6b,9,9,12a-heptamethyl-2,3,4,5,6,6a,7,8, 8a,10,11,12,13,14b-tetradecahydro-1H-picene-4a-carboxylic acid	Pygenic acid A	12.8	[M-H]^-^	C_30_H_48_O_4_	35,174,341.91
	(4aS,6aS,6bR,9S,12aR)-10,11-dihydroxy-9-(hydroxymethyl)-1,2,6a,6b,9,12a-hexamethyl-2,3,4,5,6,6a,7,8,8a,10,11,12,13, 14b-tetradecahydro-1H-picene-4a-carboxylic acid	Pygenic acid B	10.2	[M-H]^-^	C_30_H_48_O_5_	22,142,874.09
	(2S,3S,8S,9R,10R,13R,14S,16R,17R)-17-[(2R)-2,6-dihydroxy-6-methyl-3-oxoheptan-2-yl]-2,3,16-trihydroxy-4,4,9,13,14-pentamethyl-1,2,3,7,8,10,12,15,16,17-decahydrocyclo penta[a]phenanthren-11-one	Cucurbitacin IIb	12.4	[M+H]^+^	C_30_H_48_O_7_	8,799,916.04
	6-[16-(acetyloxy)-2,6,6,11,15-pentamethyl-5,9,12,17-tetra oxotetra cyclo[8.7.0.0²,⁷.0¹¹,¹⁵]heptadec-1(10)-en-14-yl]-2-methyl-4-oxoheptanoic acid	Ganoderic Acid F	14.6	[M+Na]^+^	C_32_H_42_O_9_	5,213,579.53
	3β-Hydroxyurs-12-en-28-oic acid	Ursolic Acid	14.1	[M-H]^-^	C_30_H_48_O_3_	32,702,225.99
	3β-hydroxy-11-oxoolean-12-en-30-oate	Glycyrrhetinate	5.0	[M-H]^-^	C_48_H_82_O_4_	19,682,449.99
Alkaloids and derivatives	(2E,4E)-5-(1,3-benzodioxol-5-yl)-1-piperidin-1-ylpenta-2,4-dien-1-one	Piperine	10.3	[M+H]^+^	C_17_H_19_NO_3_	10,944,769.82
	methyl (3β,16β,17α,18β,20α)-11,17-dimethoxy-18-[(3,4,5-trimethoxybenzoyl)oxy]yohimban-16-carboxylate	Reserpine	14.5	[M+H]^+^	C_33_H_40_N_2_O_9_	13,189,834.99
	(E)-5-(1,3-benzodioxol-5-yl)-1-piperidin-1-ylpent-2-en-1-one	Piperanine	10.1	[M+H]^+^	C_17_H_21_NO_3_	1,207,662.86
Stilbenes	[4-[(E)-2-(3,5-diacetyloxyphenyl)ethenyl]phenyl] acetate	Triacetyl resveratrol	3.7	[M-H]^-^	C_20_H_18_O_6_	46,791,443.43
	5-[(E)-2-(4-hydroxyphenyl)ethenyl]benzene-1,3-diol	Resveratrol	8.5	[M-H]^-^	C_14_H_12_O_3_	770,352.59
Catechols	benzene-1,2-diol	Catechol	2.8	[M-H]^-^	C_6_H_6_O_2_	9,914,359.16
Hydroxy- cinnamic acids	(E)-3-(4-hydroxyphenyl)prop-2-enoic acid	p-Coumaric acid	13.2	[M+H]^+^	C_9_H_8_O_3_	293,729.18
	(E)-3-(3,4-dihydroxyphenyl)prop-2-enoic acid	Caffeic acid	10.3	[M+H]^+^	C_9_H_8_O_4_	1,504,036.81
	Methyl (E)-3-phenylprop-2-enoate	Methyl trans-cinnamic acid	8.5	[M+H]^+^	C_10_H_10_O_2_	520,096.88
Coumarins	2H-1-benzopyran-2-one	Coumarin	12.6	[M+H]^+^	C_9_H_6_O_2_	1,443,246.73
Steroid lactones	(1S,2R,5S,9S,10S,11R,12R,13R,16R)-9,12-dihydroxy-2,6,10,16-tetramethyl-14-oxatetracyclo[11.2.1.0²,¹¹.0⁵,¹⁰] hexadec-6-ene-3,8,15-trione	Eurycomalactone	12.6	[M+H]^+^	C_19_H_24_O_6_	11,323,937.79
Hydroxy- anthraquinones	(1S,2R,3S,4R)-1,2,3,4,8-pentahydroxy-6-methoxy-3-methyl-2,4-dihydro-1H-anthracene-9,10-dione	Altersolanol A	3.7	[M+H]^+^	C_16_H_16_O_8_	10,020,554.31
Aniline and substituted anilines	2-Aminophenol	o-Aminophenol	0.6	[M-H]^-^	C_6_H_7_NO	9,860,119.88
Androgens and derivatives	(1S,3aS,3bS,5aR,9aR,9bS,11aS)-N‑[2,5‑bis(trifluoromethyl)phenyl]‑9a,11a‑dimethyl‑7‑oxo‑1,2,3,3a,3b,4,5,5a,6,9b,10,11‑dodecahydro‑indeno[5,4‑f]quinoline‑1‑carboxamide	Dutasteride	12.9	[M+H]^+^	C_27_H_30_F_6_N_2_O_2_	8,402,962.94

## Discussion

Anthracnose disease, caused by *Colletotrichum* species, is a serious problem affecting many tropical plants and results in significant crop losses, predominantly in the postharvest period when fruits are highly susceptible [2,4,29]. In this study, nine *Colletotrichum* species (*C. asianum*, *C. brasiliense*, *C. fructicola*, *C. musae*, *C. nymphaeae*, *C. okinawense*, *C. orchidearum*, *C.* *pandanicola*, and *C. truncatum*) from six species complexes were isolated from various hosts, including tropical fruits such as papaya, banana, mango, passion fruit, avocado, and chili; the temperate fruit strawberry; and the ornamental plant philodendron. Some of the isolated *Colletotrichum* species exhibited strong host preferences, such as *C. musae* in banana [30], *C. asianum* in mango [31,32], and *C. truncatum* in chili [33]. However, multiple *Colletotrichum* species have been found to infect or colonize the same host plant [34]. For instance, Fuentes-Aragón et al. [35] reported that *C. karsti, C. godetiae*, *C. siamense*, *C. fioriniae*, and *C. nymphaeae* were isolated from avocados in Mexico. In contrast, *C. fructicola* and *C. pandanicola* were isolated from avocados in the present study. Furthermore, some species such as *C. okinawense,* isolated from Holland papaya were reported for the first time in Thailand. Previously, this species had only been documented in Japan, Brazil, and Taiwan [36,37]. In this study, *C. nymphaeae* was isolated from strawberries. To date, there have been no documented reports of *C. nymphaeae* in Thailand; however, it has been identified as a pathogen in various other regions, including Brazil, South Korea, and China, affecting multiple hosts such as strawberry and walnut [38–40].

Nowadays, research on the discovery of antifungal agents from natural sources, including plants, has gained increasing attention as a sustainable strategy to control and manage anthracnose caused by *Colletotrichum* species, while also reducing the reliance on chemical fungicides. For example, extracts from allspice (*Pimenta dioica*) [41], Indian borage (*Plectranthus amboinicus*) [41], garlic (*Allium sativum*) [42], ginger (*Zingiber officinale*) [42], and camphor tree (*Cinnamomum camphora*) [43] have demonstrated antifungal activity by inhibiting the mycelial growth and spore germination of *Colletotrichum* species. In this study, ten medicinal plants including lemongrass, clove, sea holly, heart-leaved moonseed, turmeric, black pepper, cassumunar ginger, Indian gooseberry, cinnamon, and kariyat were screened for antifungal activity against nine *Colletotrichum* species at a concentration of 100 mg/mL. Among these, only the ethanolic extract of clove showed antifungal activity against all nine *Colletotrichum* species isolated in this study. Further research at increased extract concentrations is required to fully evaluate the antifungal potential of the remaining nine medicinal plants against all *Colletotrichum* species identified in this study. Previous studies have reported that clove essential oil significantly inhibited the mycelial growth and conidia germination of *C. gloeosporioides*, likely due to its ability to damage the fungal cell wall and membrane, resulting in leakage of intracellular contents [44,45]. Moreover, clove essential oil treatment has been shown to impair cell membrane integrity and biological function by downregulating genes involved in membrane components and transmembrane transport [46].

Several studies have demonstrated that the plant source, plant part, cultivation conditions, extraction solvent, and extraction method can significantly affect the chemical composition of plant extracts [47–49]. Although the chemical composition of clove has been reported previously, it was necessary to identify it in this study. Therefore, GC-MS and LC-MS analyses were performed to identify the chemical composition of the ethanolic clove extract. In this study, eugenol was identified as the predominant compound in the ethanolic clove extract via GC-MS analysis, consistent with previous reports [46,50]. To confirm the antifungal activity of eugenol, methyl eugenol (at a final concentration of 250 mg/mL) was tested, and the results showed that it inhibited all isolated *Colletotrichum* species. In addition, *β*-caryophyllene, eugenyl acetate, and *α*-humulene also detected in the clove extract have been reported to exhibit antifungal activity against phytopathogenic fungi, primarily by inhibiting hyphal growth and spore germination [51–53]. Beyond the well-known essential oil component eugenol, bioactive compounds in the clove extract were further analyzed using LC-MS in this study. Several flavonoids, including quercetin, kaempferol, isorhamnetin, and myricetin, were identified, along with phenolics such as gallic acid, ellagic acid, and catechol. Hydroxycinnamic acids, including caffeic acid, *p*-coumaric acid, and methyl trans-cinnamic acid, as well as stilbenes like resveratrol, were also detected in the ethanolic clove extract, consistent with previous reports [54,55]. These flavonoids and phenolic compounds have demonstrated notable antifungal properties against phytopathogenic fungi such as *Fusarium*, *C. gloeosporioides*, *Botrytis cinerea,* and *Alternaria alternata* [56–59]. Their mechanisms of action typically involved disrupting fungal cell walls, compromising membrane integrity, and inhibiting essential fungal enzymes. Additionally, they interfere with organic acid secretion and the biosynthesis of mycotoxins such as fumonisin B1 and beauvericin produced by *Fusarium*. Moreover, bioactive compounds from the triterpenoids group such as pygenic acid, cucurbitacin, ganoderic acid, and ursolic acid as well as alkaloids and their derivatives, including piperine, reserpine, and piperanine, were identified in the clove phytoextract in this study. These compounds have also been reported in other medicinal plants in previous studies [60–64]. Triterpenoids and alkaloids found in plants exhibit antifungal, antibacterial, and antioxidant activities, as well as anticancer and anti-inflammatory properties [60,65]. However, antifungal activity observed *in vitro* may not fully reflect *in vivo* responses due to environmental factors and plant metabolic processes. Therefore, future studies will involve controlled *in vivo* assays on fruit and plant tissues to evaluate the efficacy of the ethanolic clove extract in controlling anthracnose disease caused by *Colletotrichum* species. Lesion development, spore germination, and disease progression will then be monitored to validate the *in vitro* findings. Furthermore, future studies will determine a suitable control concentration that is non-toxic to the target fruits and plants.

## Conclusion

This study highlighted the diversity of *Colletotrichum* species associated with fruits and ornamental plants in Thailand, identifying eleven isolates across nine species: *C. asianum*, *C. brasiliense*, *C. fructicola*, *C. musae*, *C. nymphaeae*, *C. okinawense*, *C. orchidearum*, *C. pandanicola*, and *C. truncatum*. The findings also demonstrate the promising antifungal potential of clove ethanolic extract, which showed strong inhibitory activity against all isolated *Colletotrichum* species, with MICs ranging from 12.5 to 25 mg/mL. GC-MS and LC-MS analyses revealed a rich profile of bioactive compounds in the clove extract, including eugenol, flavonoids, phenolics, terpenoids, and alkaloids. These results support the potential use of clove phytoextracts as a natural and sustainable alternative for fungal management in agriculture. Overall, the findings of this study enhance our understanding of *Colletotrichum* species associated with fruits and ornamental plants in Thailand and offer valuable insights for developing effective alternative management strategies using plant-based extracts.
